# 

**Published:** 1860-10

**Authors:** 


### CONTENTS OF HALL'S JOURNAL OF HEALTH FOR OCTOBER.

(New-York, $1 a year.)

OUR BOYS, ........ 217
MANUAL LABOR SCHOOLS, ........ 219
SMALL POX, ........ 221
NERVOUSNESS, ........ 222
LIFE'S MAXIMS, ........ 223
"SNAPPING UP," ........ 225
WARMING HOUSES, ........ 226
CHILDREN'S EATING, ........ 228
MILK, ........ 230
REARING CHILDREN, ........ 231
OVER-EATING, ........ 232
APPLES, ........ 233
ELECTRO-MAGNETISM, ........ 234
WEARING GARTERS, ETC., ETC., ........ 234

